# Animal models of Soft Tissue Sarcoma for alternative anticancer therapy studies: characterization of the A-72 Canine Cell Line

**DOI:** 10.1007/s11259-023-10115-z

**Published:** 2023-04-11

**Authors:** Elisabetta Razzuoli, Barbara Chirullo, Chiara Grazia De Ciucis, Samanta Mecocci, Isabella Martini, Roberto Zoccola, Chiara Campanella, Katia Varello, Paola Petrucci, Antonio Di Meo, Elena Bozzetta, Michela Tarantino, Maria Goria, Paola Modesto

**Affiliations:** 1 National Reference Center of Veterinary and Comparative Oncology (CEROVEC), Istituto Zooprofilattico Sperimentale del Piemonte, Liguria E Valle D’Aosta, Piazza Borgo Pila 39/24, 16129 Genoa, Italy; 2 Unit of Emerging Zoonoses Department of Food Safety, Nutrition and Veterinary Public Health, Istituto Superiore Di Sanità, Viale Regina Elena 299, 00161 Rome, Italy; 3 Department of Veterinary Medicine, University of Perugia, Via San Costanzo 4, 06126 Perugia, Italy

**Keywords:** A-72, Soft tissue sarcoma, Gene expression, Innate immunity, *Salmonella Typhimurium*

## Abstract

Canine Soft Tissue Sarcoma (STS) cell line A-72 has been largely employed for antiviral and antiproliferative studies. However, there are few information on their characteristics. Our aim was to evaluate A-72 expression level of genes and proteins involved in the innate immune response and cell cycle, their ability to respond to infective stressors and their possible use as a cellular model for anti-cancer studies in human and animal medicine. For this purpose, we evaluated the basal expression of immune-related, cell cycle and DNA repair genes on this cell line and tumoral tissues. A-72 ability to respond to a wild-type strain of *Salmonella typhimurium* was assessed. *S. typhimurium* showed ability to penetrate A-72 causing pro-inflammatory response accompanied by a decrease of cell viability. *IL10* and *IL18* genes were not expressed in A-72 while *CXCL8*, *NOS2*, *CXCR4* and *PTEN* were highly expressed in all samples and *TP53* was slightly expressed, as shown in human STS. Our results outline the ability of A-72 to respond to a bacterial agent by modifying the expression of important genes involved in innate immune response and provide a useful model for in vitro evaluation of new therapeutic approaches that could be translated into the human oncology.

## Introduction

Soft tissue sarcoma (STS) is a term used both in human and animal oncology to describe different types of cancer that can arise from many anatomical sites. Despite their widespread dissemination into the organism, these tumors have a common mesenchymal origin and have similar biological and histological features (Dennis et al. 2011). Tumors that are included in the STS group are: perivascular wall tumors, liposarcoma, malignant fibrous histiocytoma, mesenchymoma, myxosarcoma, non-plexus derived peripheral nerve sheath tumors, undifferentiated sarcoma and fibrosarcoma (Bray 2016). These cancers are usually described as pseudo-encapsulated masses with poorly defined margins and malignant tumor cells have the ability to exit the cancer core penetrating through the pseudo-capsule. Consequently, after the surgical resection, a microscopic tissue residue in situ can remain, resulting in tumor recurrence (Bray 2017). In humans, through rare (about 1% in adults), STS are highly debilitating malignancies and are often associated with significant morbidity and mortality; the standard of care for localized disease in adults has been wide surgical resection (Dodd et al. 2010; Gamboa et al. 2020). The rarity and heterogeneity of patient samples (about 100 different histologic and molecular sub-types of STS are recognized) complicate clinical investigations into STS biology (Dodd et al. 2010; Gamboa et al. 2020). Sarcomas research relied on human cell lines and immunocompromised mice. Some human cell lines derived from STS are available and have also been used as xenografts in immunocompromised mice in order to study anticancer drug development, drug sensitivity and treatments response (Dodd et al. 2010). In dogs, if a complete tumor surgery resection is possible, prognosis is good (Bray et al. 2014). Today it is accepted that radical excision margins (about 2–3 cm) will provide good local cancer control (Bray 2016); however, no diagnostic tests are currently available to predict the correct dimension of surgical margin required for each tumor, so relapse can develop between 17 and 75% of dogs and is associated with increased risk of death (Bray 2017).

Despite many advances in elucidating progression, invasiveness and metastatic spread of STSs, the use of in vitro sarcomas models has led to relatively few new drug treatments and according to current literature, surgical resection is still the treatment of choice to achieve long-term disease-free survival or a cure both in humans and dogs (Ettinger 2003; Selting et al. 2005; Hager et al. 2017). Dogs and humans STSs display similar histological and immunohistochemical features (Milovancev et al. 2015), therefore, there is great interest in animal models of primary tumors to elucidate STS biology underlying their development, progression, and treatment (Dodd et al. 2010). From this point of view, the availability of STS animal cell cultures can be useful in order to verify the efficacy of treatments to be transferred first to the veterinary and then to human research against cancer.

In dogs, radiation therapy plays an important role in the management of STSs, but it has little role as a single treatment modality. Radiation therapy is appropriate for incompletely excised tumors or for preoperative treatment. Due to the generally poor response of the STS to chemotherapy, it has a role as additional treatment to treat incompletely resected tumors, high-grade tumors, and metastatic disease or as palliative therapy in unresectable tumors (Ettinger 2003). Doxorubicin-containing protocols and mitoxantrone are the most effective drugs against canine STS, with reported response rates of approximately 20% (Ettinger 2003; Selting et al. 2005). In humans, radiotherapy in addition to surgery has shown to enhance local control and resectability in some kind of STSs (Hager et al. 2017). Several agents have been investigated for chemotherapy in human and, to date, doxorubicin and ifosfamide remain the most effective chemotherapy drugs available for the treatment of majority of these tumors (Liu et al. 2018; Ratan and Patel 2016).

Under this point of view, multimodal therapies based on the association of surgery with radiotherapy/chemotherapy/immunotherapy/electrochemotherapy could represent an alternative therapeutic strategy (Spugnini et al. 2019; Torrigiani et al 2019); this could determine a reduction in surgical excision and STS recurrence. A recent study suggests that chemotherapy causes a decrease of tumor growth by immune-modulatory effects (Bajpai and Susan 2016). A promising strategy could be to develop new anti-STS therapy based on immune response modulation using attenuated bacteria (Chirullo et al. 2015a, b).

In this respect, cell lines could be helpful to test in vitro immunomodulant anticancer therapies, allowing to reduce the use of laboratory animals in the preliminary tests and to achieve results in shorter time. In this context, it is pivotal to develop and evaluate in vitro models of dog-STS, furthermore, the evaluation of the suitability of the canine spontaneous STS as a model for the study of the human STS is of great interest. However, few and recent STS cell lines are available in dog (Snyder et al. 2011; Son et al. 2019). Moreover, to the authors’ knowledge, these cells lines have not been characterized in terms of gene expression and their ability to interact with infective stressors is unknown. In this framework, the canine tumor cell line A-72 was obtained by Binn and co-workers in 1980 (Binn et al. 1980) from a tumor of 1 cm diameter taken from the left thigh of an eight years old female Golden Retriever. After the explant, cells were serially passaged, and they maintained a fibroblastic appearance (Binn et al. 1980). Since then, many researchers have used A-72 as cell line for in vitro test on viruses (Martin-Calvo et al. 1994; Pratelli and Moschidou 2012; Parthiban et al. 2014), vaccines (Barros et al. 2017), antiviral and anti-proliferative compounds (Croci et al. 2011; Fan et al. 2014). However, little is known about the A-72 cells in terms of the origin phenotype, expression of genes involved in the innate immune response, DNA repairs and cell cycle regulation. The aim of our study was to characterize A-72 cells first evaluating the basal expression level of genes and proteins involved in the innate immune response and in the cell cycle; secondly assessing their modulation induced by a bacterial agent (specifically *S. Typhimurium)*, in order to evaluate the ability of cells to respond to this stressor. *S. Typhimurium* is the second most frequent serotype of *Salmonella* isolated in Europe from both humans and veterinary matrices and represents the most prevalent and disseminated serovar worldwide (Ferrari et al. 2019)and its attenuated form was deeply investigated for its antitumor activity in human and murine cells (Chirullo et al. 2015a).

## Materials and methods

### Cell culture

A-72 cells line (canine tumor, IZSLER biobank OIE codex BS TCL 1) at 98^th^ passage were purchased and used for different experiments. A-72 cells were grown in a mixture of Eagle’s Minimum Essential Medium Eagle (Merck, Milan, Italy, cat M5650) enriched with Sodium Pyruvate 1% (Euroclone, Milan, Italy, cat ECM0542D), 10% (v/v) of Fetal Bovine Serum (FBS, GIBCO™, Thermofisher Scientific, Milan, Italy, cat 10,437–036) and a mixture of Antibiotics (penicillin and streptomycin, 1% v/v, Carlo Erba Reagents, cat FA30WL0022100). For the experiments, 12-well culture plates (2.5 × 10^5^ cells/ml, 2 ml per well) were incubated at 37 °C in 5% CO_2_ until confluence (16–22 h). Cells were tested at the 104^th^, 107^th^ and 109^th^ passages as described in Sect. "Gene Expression"; to evaluate basal gene expression. Moreover, cells, at the 109^th^ passage cells were incubated for 24 ± 1 h (hrs) after confluence to evaluate the effects of monolayer aging. Each experiment was repeated six times.

### Gene expression

Gene expression was evaluated in A-72 cell line at 104^th^, 107^th^ and 109^th^ passages and in one neoplastic sample (hemangiosarcoma-HE) obtained during the routinely surgery and stored in RNAlater (Machery-Nagel GmbH & Co KG, Durren, Germany, cat 740,400,500). Surgery samples were obtained by the University of Perugia, Department of Veterinary Medicine.

Gene selection been made based on their role in innate immune response to microorganisms. Moreover, we considered their involvement in STS development and metastasis (Chirullo et al. 2015b; Razzuoli et al. 2017; Li et al. 2017; Gustafson et al. 2018). The following genes were tested: Interleukins (IL) (*IL6*, *CXCL8*, *IL10*, *IL15*, *IL18)*, Nitric oxide synthase 2 (*NOS2)*, Cluster of Differentiation 14 and 44 (*CD14* and *CD44)*, C-X-C chemokine receptor type 4 (*CXCR4)*, Erb-B2 Receptor Tyrosine Kinase 2 (*ERBB2)*, Lymphocyte antigen 96 (*LY96)*, Myeloid differentiation primary response 88 (*MYD88)*, Transforming growth factor beta (*TGFB),* Nuclear factor kappa-light-chain-enhancer of activated B cells (*NFKB/p65)*, Phosphatase and tensin homolog (*PTEN)*, Signal transducer and activator of transcription 5 (*STAT5)*, Toll-like receptor 4 and 5 (*TLR4* and *TLR5),* onco-suppressor *TP53* and DNA repair *RAD51*; Ribosomal protein *S5 (RPS5)* was used as reference gene. To this purpose, we used primers sets described in previous works (Table 1). Total RNA extraction from A-72 cells and tumors was carried out by the RNeasy Mini Kit (Qiagen s.r.l., Milan Italy, cat 74,104) on the QiaCube instruments (Qiagen s.r.l.) according to the manufacturer’s instructions, reverse transcription (RT) was performed with the OneScript® cDNA Syntesis Kit (Applied Biological Materials Inc. Richmond, BC, Canada, cat G234) using 250 ng of total RNA. The gene expression was assessed by RT-qPCR using primers reported in Table 1 and the Sybr Green chemical approach (Applied Biological Materials Inc., cat 4,367,659) using our protocols as previously described (Razzuoli et al. 2014). RT and Real-Time PCR amplification were performed on a CFX96™ Real-Time System (Bio-Rad, Milan, Italy). To evaluate the basal expression level in each sample, the relative expression of the target genes was calculated using the formula 2^−ΔΔCq^. The mean of three test replicates ± 1 standard deviation was considered. To identify PCR-negative samples was chosen a Cq value of 39.

**Table 1:** Primer Sets for Sybr Green quantitative, Real-Time RT-PCR amplification of canine genes

Genes	Primer Sets		Length (bp)	number of accession	References
*CD14*	Forward	GCCGGGCCTCAAGGTACT	60	XM_843653.4	(Silva et al. 2010)
	Reverse	TCGTGCGCAGGAAAAAGC			
*CD44*	Forward	CAAGGCTTTCAACAGCACCC	191	NM_001197022.1	(Capellini et al. 2020)
	Reverse	TACGTGTCGTACTGGGAGGT			
*CXCR4*	Forward	GCGTCTGGATACCTGCTCTC	163	NM_001048026.1	(Capellini et al. 2020)
	Reverse	GATACCCGGCAGGATAAGGC			
*ERBB2*	Forward	CTGAGGGCCGATATACCTTC	113	NM_001003217.2	(da Costa et al. 2011)
	Reverse	TCACCTCTTGGTTGTTCAGG			
*IL6*	Forward	TCCAGAACAACTATGAGGGTGA	99	NM_001003300.1	(Cavalcanti et al. 2015)
	Reverse	TCCTGATTCTTTACCTTGCTCTT			
*CXCL8*	Forward	TGATTGACAGTGGCCCACATTGTG	355	D14285.1	(Capellini et al. 2020)
	Reverse	GTCCAGGCACACCTCATTTC			
*IL10*	Forward	CGACCCAGACATCAAGAACC	100	NM_001003077.1	(Peeters et al. 2005)
	Reverse	CACAGGGAAGAAATCGGTGA			
*IL15*	Forward	ACTTGCATCCAGTGCTACTT	270	AF479882.1	(Choi et al. 2006)
	Reverse	CGAGCGAGATAACACCTAAC			
*IL18*	Forward	CTCTCCTGTAAGAACAAAACTATTTCCTT	99	NM_001003169.1	(Kurata K et al. 2004)
	Reverse	GAACACTTCTCTGAAAGAATATGATGTCA			
*NOS2*	Forward	AGACACACTTCACCACAAGG	284	AF07782.1	(Kaim et al. 2006)
	Reverse	TGCTTGGTGGCGAAGATGAGC			
*LY96*	Forward	GGGAATACGATTTTCTAAGGGACAA	91	XM_848045.3	(Silva et al. 2010)
	Reverse	CGGTAAAATTCAAACAAAAGAGCTT			
*MYD88*	Forward	GAGGAGATGGGCTTCGAGTA	159	XM_534223.5	(Capellini et al. 2020)
	Reverse	GTTCCACCAACACGTCGTC			
*NFKB/p65*	Forward	TGTAAAGAAGCGGGACCTGG	249	AB930129.1	(Ishikawa et al. 2015)
	Reverse	AGAGTTTCGGTTCACTCGGC			
*PTEN*	Forward	GTGAAGCTGTACTTCACAA	135	NM_001003192.1	(Kanae et al. 2006)
	Reverse	CTGGGTCAGAGTCAGTGGTG			
*TP53*	Forward	CGTTTGGGGTTCCTGCATTC	231	NM_001003210.1	(Capellini et al. 2020)
	Reverse	CACTACTGTCAGAGCAGCGT			
*RAD51*	Forward	GGAGAAGGAAAGGCCATGTA	147	NM_001003043.1	(Klopfleish et al. 2009)
	Reverse	GGGTCTGGTGGTCTGTGTT			
*STAT5*	Forward	TTGACTCTCCTGACCGCAAC	181	XM_548091.5	(Capellini et al. 2020)
	Reverse	TCCGTCTACTGCTTTAGCGA			
*TGFB*	Forward	CAAGTAGACATTAACGGGTTCAGTTC	70	L34956	(Maissen-Villiger et al. 2016)
	Reverse	GGTCGGTTCATGCCATGAAT			
*TLR4*	Forward	GCTGGATGGTAAACCGTGGA	157	NM_001002950.2	(Capellini et al. 2020)
	Reverse	AGCACAGTGGCAGGTACATC			
*TLR5*	Forward	CCAGGACCAGACGTTCAGAT	108	EU551146.1	(Turchetti et al. 2015)
	Reverse	GCCCAGGAAGATGGTGTCTA			
*RPS5*	Forward	TCACTGGTGAGAACCCCCT	141	XM_533568	(Selvarajah et al. 2017)
	Reverse	CCTGATTCACACGGCGTAG			

### Immunocytochemical assay

The following antibodies were used for the Immunocytochemical analysis: Polyclonal Rabbit CXCR4 (Sigma Aldrich, cat C3116) and S100 (Dako Denmark A/S, cat Z0311); Monoclonal Mouse Actin (ACT, Clone 1A4, Dako Denmark A/S, cat M0851), Calponin (CALP, Clone CALP, Dako Cytomation, cat M3556), CD44 (Clone DF1485, Dako Denmark A/S, cat M7082), Cytokeratin (CK, Clones AE1/AE3, Dako North America, Inc., cat M3515), Desmin (DESM, Clone D33, Dako Denmark A/S, cat M0760), and Vimentin (VIM, Clone V9, Dako Denmark A/S, cat M0725). 1% of Bovine Serum Albumin (BSA, Merck, Milano Italy, cat A3418) in Phosphate Buffered Saline was used for dilution (PBS, Merck, Milano Italy, cat. D8537). The dilutions were performed with 1% BSA (Applichem GmbH, Darmstadt, cat A1391) in PBS.

A-72 cells (2.5 × 10^5^ cells/ml) were treated with the primary antibody diluted as follow: CK 1:50 for 10 min (mins), VIM 1:200 for 30 min, ACT and DESM 1:100 for 30 min, CALP and S100 1:400 for 30 min, CD44 1:7 and CXCR4 1:500 overnight (Modesto et al. 2020). Afterward, samples were washed three times for 5 min with PBS and treated with the secondary antibody (Dako EnVision^+^ Dual Link System-HRP, Dako North America, Inc. cat K4061): ACT and DESM for 1 h, CXCR4 for 50 min, CALP, CD44, CK, and VIM for 30 min and S100 for 20 min. Then, cells were washed three times for 5 min with PBS and the chromogenic fixation was carried out with 3,3’-diaminobenzidine (DAB, Dako Liquid DAB + Substrate Chromogen System, Dako North America, Inc. cat K3468); the reaction was blocked in distilled water for 5 min. Cells incubated with the immunoglobulin fraction of the mouse non-immune serum, instead of the primary antibody, were used as negative control. Finally, the slides were controstained with Mayer’s hematoxylin (Sigma Aldrich, cat MHS1) and observed under a light microscope.

### Response to infective stressor

To evaluate the ability of this cell line to respond to infective stressor we used a wild-type strain of *S. Typhimurium* (*ST*) according to our previous study (Modesto et al. 2020). *ST* was grown to obtain mid-log phase culture. *ST* was re-suspended at 10^8^ colony forming unit (CFU)/ml, and A-72 cells were treated with 1 ml of this bacterial suspension (MOI 100:1 CFU/cells) for 1 h at 37 °C in 5% CO_2_. Cells treated were used to assess the *ST* stimulation effects through the evaluation of 1) immunomodulation, 2) cells vitality and 3) invasiveness.

#### Experiment 1: Modulation of innate immune response

After A-72 treatment with *ST* and incubation at 37 °C in 5% CO_2_ for 1 h, cells were washed three times with medium only and incubated at 37 °C in 5% CO_2_ for 3 h with fresh completed medium. The experiment was repeated three times and cells treated exclusively with medium without bacteria suspension were used as negative control. The gene expression analysis was carried out as described in 2.2 section.

#### Experiment 2: Cell vitality after treatment

After 1 h of *ST* exposure cells were washed, detached with trypsin (Sigma Aldrich, Saint Louis, Missouri, USA, cat T4049) and blocked with complete medium; after this, 10 μl of Trypan Blue 0.4% (Logos Biosystems, Inc. Korea, cat. T13001) were added to 10 μl of cell suspension to evaluate cell viability using the LUNA II Automated Cell Counter (LUNA™ Logos Biosystems, Inc. Korea). As negative control cells treated with medium only were used. Experiment was repeated thrice.

#### Experiment 3: Bacterial invasion

Cells at confluence were infected with 1 ml of bacterial suspension (see Sect. "Response to Infective Stressor") at 10^8^ CFU/ml and incubated at 37 °C in 5% CO_2_ for 1 h in agreement with the protocols described by Razzuoli et al. (Razzuoli et al. 2017). Briefly, after *ST* exposure, cells were washed with medium and treated with a solution of PBS containing 300 μg/ml colistin sulphate (Microbiol & C. s.n.c., Cagliari, Italy, cat 74,016) at 37 °C in 5% CO_2_ for 2 h to remove all extracellular bacteria. Absence of toxic side effects on A-72 had been confirmed in preliminary assays. Then, cells were lysed adding 1% Triton X-100 (Sigma Aldrich, Saint Louis, Missouri, USA, cat 108,603) in PBS at room temperature for 5 min. Afterwards, PBS was added to each well; the resulting cell suspension was vortexed, serially diluted and seeded on Xylose Lysine Deoxycholate (XLD; Sigma Aldrich, cat 146,073) agar plates and incubated at 37 °C for 24–48 h. Cells treated with medium only were used as negative control; the experiment was performed thrice.

### Sequencing of the key gene CXCR4 and TP53

The presence of mutations that can modify CXCR4 and TP53 expression has been investigated by direct sequencing in A-72 cancer cell line and in canine healthy tissue samples and we compared the obtained sequences with those deposited in the GeneBank database. Tests were performed on A-72 (at 98^th^ and 109^th^ passages) and on 5 healthy canine tissues (2 lymphonodes, 2 kidney and 1 liver), three replications for each experiment have been carried out. DNA was extracted from 1 × 10^6^ cells and 25 mg of each healthy tissue using QIAmp DNA Mini kit (Qiagen, Milan, Italy, cat 51,306) following the manufacturer’s protocol.

Sequencing of TP53 was carried out using primer pair (dogP53F2 5'-CTC CTC AGC ATC TCA TCC G-3' and dog P53R2 5'- ATG GCG AGA GGT AGA TTG C -3') specifically designed to amplify a 2000 pb fragment of the TP53 (reference sequence ID KJ511265.1). Sequencing of CXCR4 was performed as previously described (Modesto et al. 2020). Specific primers pair spanning a 902 bp of the coding region have been designed on the reference sequence (NM_001048026.1) (Table 2).

**Table 2:** Primers designed in this paper for the amplification and sequencing of canine *CXCR4*

Primer		Position	Product lenght (bp)	Accession number	Source
*CXCR4 F1*	TGACTCCATGAAGGAACCCTG	F 88–108 R 971–990	902	NM_001048026.1	This paper
*CXCR4 R1*	CTGCTCACAGAGGTGAGTGC				
*CXCR4 F2*	GTCATCCTGTCCTGCTACTG	F 665–684 R 296–314	Sequencing 902 bp	NM_001048026.1	This Paper
*CXCR4 R2*	CAACTGCCCAGAAGGGAAG				

All primer pairs were designed using Primer3 software version 0.4.0 (https://bioinfo.ut.ee/primer3-0.4.0). PCR reaction mix has been setted as follows: 1X PCR Buffer, 1 mM MgCl_2_, 1.25 U hot start Taq polymerase, dNTPs mix 10 mM each, 0.25 μl of each primer (Thermo Scientific, Monza, Italy). Thermal profile was 94 °C for 15 min, 35 cycles of 94 °C for 30 s (s), 60 °C for 30 s (for the CXCR4 amplification) or 58 °C for 45 s (for TP53 amplification) and 72 °C for 60 s, a final step at 72 °C for 10 min was included. A negative control was added to each run. The amplification runs were performed on a GeneAmp9700 (Applied Biosystems, Monza, Italy). The products of amplification were purified using High Pure PCR Product Purification Kit (Roche Diagnostics, Monza, Italy, cat 11,732,668,001). Sequencing reaction products were purified using the DyeEx 2.0 Spin Kit (Qiagen, Milan, Italy, cat 63,204) and a capillary electrophoresis was run on the Applied Biosystems 3500 genetic Analyzer (Thermo Scientific, Monza, Italy). The consensus sequence of the entire 902 bp segment has been obtained by the forward and reverse overlapping sequences aligned with BioEdit Sequence Alignment Editor version 7.2.5. Consensus sequences were aligned with reference sequences using the ClustalW multiple alignment function.

### Statistical analyses

Data sets were submitted to a Kolmogorov–Smirnov test to check Gaussian distributions; significant differences within normal distributions were checked by one-way ANOVA or *Student’s t* test. The significance threshold was set at *P* < 0.05 (Prism 5, GraphPad Software).

## Results

### Basal gene expression

All target genes under study have been found expressed in A-72 with the exception of *IL10* and *IL18* (Table 3). In particular, *CD44*, *CXCR4*, *ERBB2*, *IL6*, *CXCL8*, *NOS2*, *LY96*, *MYD88*, *NFKB/p65*, *PTEN*, TP*53*, *RAD51*, *STAT5*, *TGFB* and *TLR5* expression was observed in all tested samples. Concerning the other genes, *CD14* was expressed in 72.2% of samples, *TLR4* in 38.9% and *IL15* in 5.5%. Moreover, same analysis were performed on sarcoma samples provided by the Department of Veterinary Medicine of University of Perugia. These tumoral tissue showed the expression of all the genes under study with the exception of *PTEN* (Table 3).

**Table 3:** Basal gene expression in A-72 cells and a neoplastic primary tissue (Hemangiosarcoma). Data are expressed as: + all samples were positive;—all samples were negative; ± only some samples were positive; ND: not detected. ΔCq = Cq (target gene)—Cq (reference gene) S.D.: standard deviation

Gene	A-72	ΔCq + S.D	Neoplastic tissue (HE)	ΔCq + S.D
*CD14*	** ± **	13.8 ± 2.0	** + **	1.5 ± 1.1
*CD44*	** + **	1.9 ± 0.9	** + **	0.9 ± 0.7
*CXCR4*	** + **	8.2 ± 1.8	** + **	4.6 ± 1.7
*ERBB2*	** + **	2.9 ± 1.3	** + **	5.8 ± 0.9
*IL6*	** + **	10.8 ± 1.7	** + **	2.8 ± 1.9
*IL8*	** + **	3.2 ± 3.3	** + **	0.3 ± 1.8
*IL10*	**-**	ND	** + **	4.9 ± 2.0
*IL15*	** ± **	15.7 ± 1.3	** + **	5.3 ± 2.2
*IL18*	**-**	ND	** + **	2.8 ± 1.8
*NOS2*	** + **	7.3 ± 1.5	** + **	2.7 ± 2.2
*LY96*	** + **	2.8 ± 1.7	** + **	1.9 ± 1.4
*MYD88*	** + **	5.1 ± 0.7	** + **	6.0 ± 0.8
*NFKB/p65*	** + **	0.3 ± 1.4	** + **	0.9 ± 1.2
*PTEN*	** + **	11.8 ± 2.4	**-**	ND
*TP53*	** + **	0.8 ± 1.3	** + **	1.2 ± 0.3
*RAD51*	** + **	2.8 ± 0.4	** + **	5.2 ± 1.8
*STAT5*	** + **	2.8 ± 2.5	** + **	1.5 ± 0.9
*TGFβ*	** + **	2.2 ± 2.1	** + **	0.6 ± 0.2
*TLR4*	** ± **	13.8 ± 2.4	** + **	3.1 ± 1.1
*TLR5*	** + **	8.4 ± 2.7	** + **	12.5 ± 1.6

### Modulation of gene expression at different cell passages and effect of monolayer aging

After basal gene expression analysis, we focused on the modulation of the gene expression at three different passages. Our results showed modulation of gene expression after 107^th^ and 109^th^ passages (Table 4) with respect to the 104^th^ passage. At passage 107^th^ we observed a significant up-regulation of *CXCR4* (*P* < 0.0001), *LY96* (*P* < 0.0001), *MYD88* (*P* = 0.0040), *TLR5* (*P* = 0.0098) and *TGFβ* (*P* = 0.00111) and significant down-regulation (defined by a value less than 1) of *IL6* (*P* = 0.00047) and *CXCL8* (*P* < 0.0001). Regarding the 109^th^ passage, we observed a significant gene expression decrease of *CD44* (*P* = 0.0401), *IL6* (*P* = 0.035), *CXCL8* (*P* = 0.0002), *NFKB/p65* (*P* = 0.00017), *TP53* (*P* = 0.00023), *RAD51* (*P* = 0.0159) and *TLR5* (*P* = 0.0076) (Table 4).

**Table 4:** Gene expression in A-72 cells at different passages. Data are expressed as 2^−ΔΔCq^ where ΔΔCq = ΔCq (107^th^ or 109^th^ passages)—ΔCq (104^th^ passage considered as basal expression). ND Not Detected. Asterisks indicate significant differences between 104^th^ passage and 107^th^ passage or 104^th^ passage and 109^th^ passages: * *P* < 0.05, **P < 0.01, ***P < 0.001 and ****P < 0.0001

Gene	107^th^ passage	109^th^ passage
*CD14*	3.11	3.71
*CD44*	1.79	0.49^*^
*CXCR4*	9.43^****^	0.69
*ERBB2*	3.24	0.74
*IL6*	0.08^***^	0.34^*^
*CXCL8*	0.02^****^	0.01^****^
*IL10*	ND	ND
*IL15*	2.66	3.05
*IL18*	ND	ND
*NOS2*	1.54	0.44
*LY96*	11.54^****^	1.16
*MYD88*	2.02^**^	0.87
*NFKB/p65*	0.95	0.14^****^
*PTEN*	0.82	0.21
*TP53*	0.96	0.15^****^
*RAD51*	1.17	0.72^*^
*STAT5*	1.27	0.62
*TGFB*	11.06^**^	0.81
*TLR4*	0.52	1.31
*TLR5*	10.13^**^	0.14^**^

**Fig. 1:**
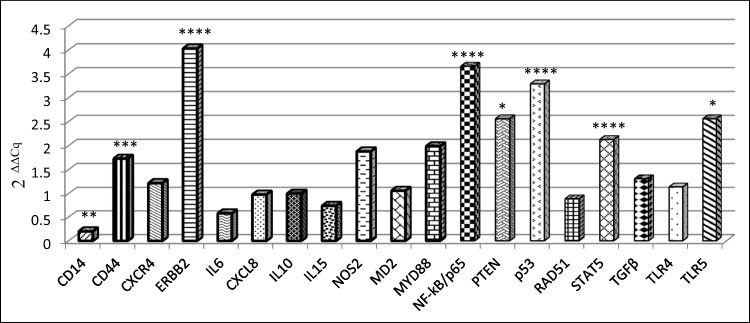
Aging effect on A-72 gene expression. Data are expressed as 2^−ΔΔCq^ where ΔCq = Cq (target gene)—Cq (reference gene); values are the mean of three test replicates ± 1 standard deviation and ΔΔCq = ΔCq (109^th^ passage at confluence plus 24 h of incubation at 37 °C)—ΔCq (control 109.^th^ passage at confluence). Negative samples were given a Cq 39 fictitious value. Asterisks indicate significant differences between control and aging cells evaluated by Student's t-test: *P < 0.05, **P < 0.01, ***P < 0.001 and ****P < 0.0001

After 24 h of monolayer achievement (109^th^ passage), cells showed a decrease of *CD14* (*P* = 0.0069), and the up-regulation of *CD44* (*P* = 0.0005), *ERBB2* (*P* < 0.0001), *NFKB/p65* (*P* < 0.0001), *PTEN* (*P* = 0.046), TP*53* (*P* = 0.00023), *STAT5* and *TLR5* (*P* = 0.0076), while the other genes were not modulated (Fig. 1).

### Immunocytochemical assay

**Fig. 2:**
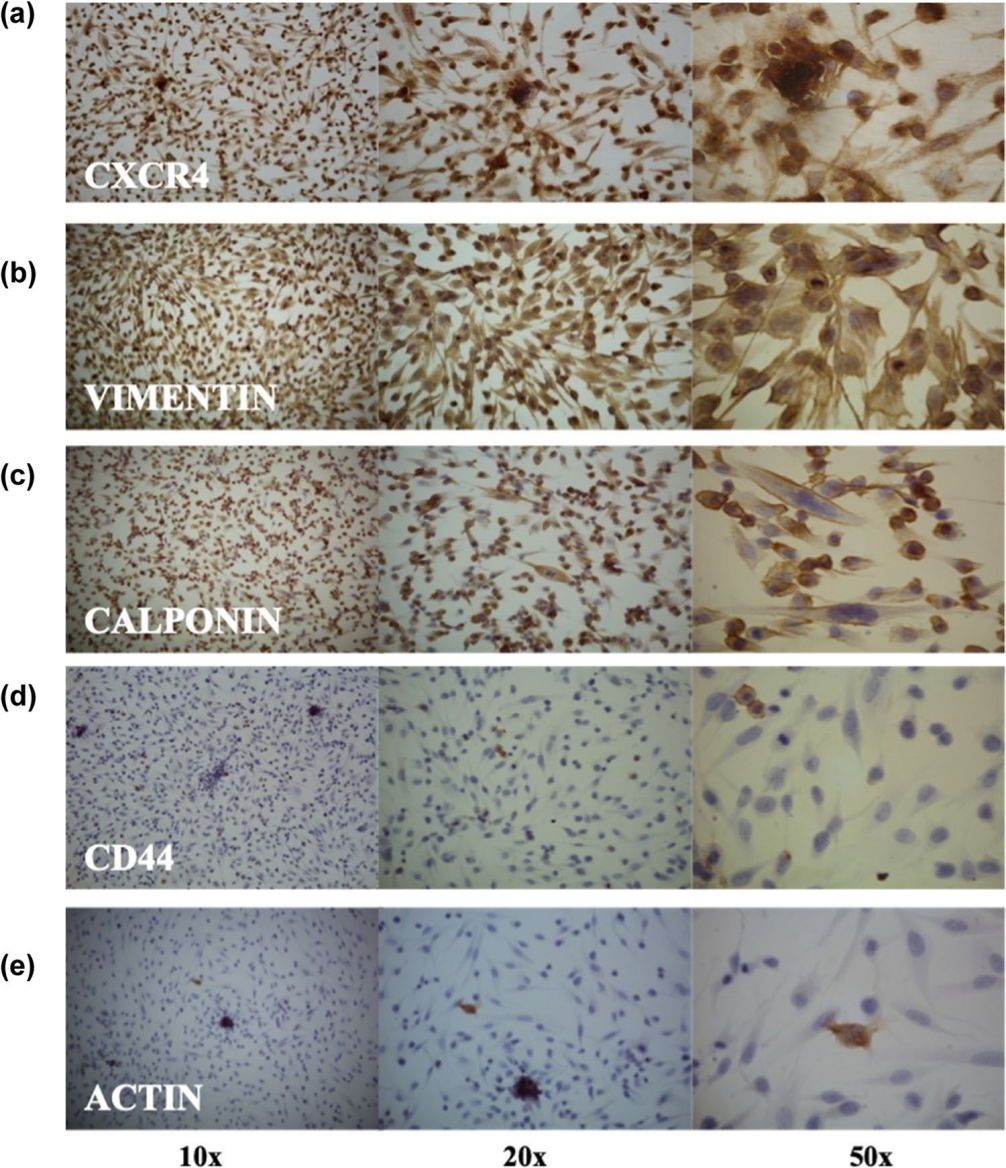
Immunocytochemical characterization of A-72 cells. Representative images of CXCR4 expression (panel **a**), VIM (panel **b**), CALP (panel **c**), CD44 (panel **d**) and ACT (panel **e**). Different magnifications are depicted

To characterize A-72 cells, immunocytochemical analyses were performed using CXCR4, VIM, CALP, DESM, S100 and CK cellular markers. Cells, at 109^th^ passages, resulted strongly positive for CXCR4, VIM and CALP (Fig. 2a, b and c); moreover, we obtained weak positivity for CD44 and ACTIN (Fig. 2d and e) while DESM, S100 and CK were negative.

### Response to infective stressor: interaction between A-72 and *ST*

**Fig. 3:**
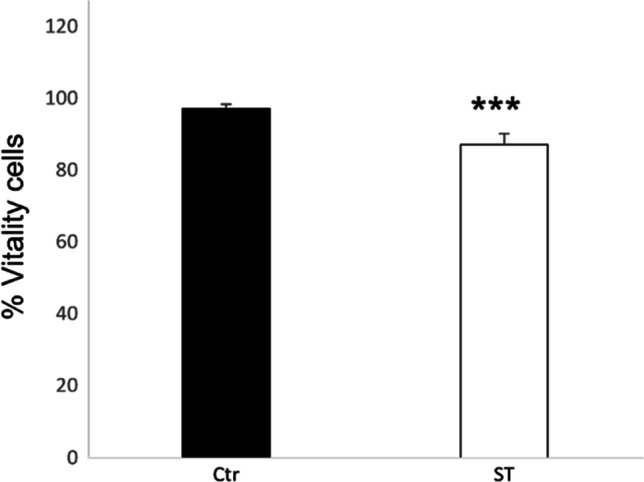
Effect of *ST* penetration on cell viability. Data are expressed as percentage of viable cells. Stars indicate significant differences between controls and ST infected cells. ***P < 0.001. Ctr: control; ST cell treated with *S. Typhimurium*

**Fig. 4:**
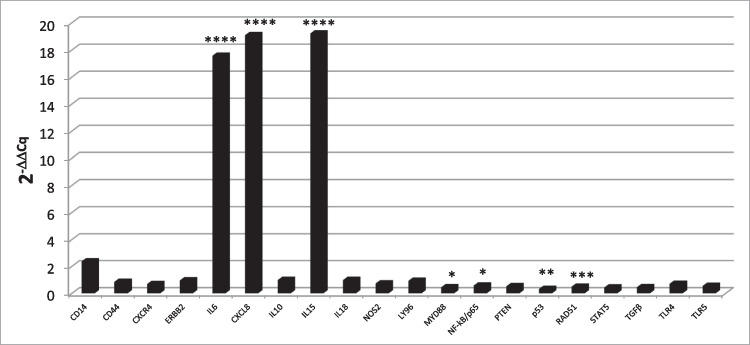
Response to Infective Stressor. Modulation of Gene expression caused by A-72 and ST interaction. Data are expressed as 2^−^.^ΔΔCq^. Negative samples were given a Cq 39 fictitious value. Asterisks indicate significant differences between controls and different passages cells: *P < 0.05, **P < 0.01, ***P < 0.001 and ****P < 0.0001

The second aim of our study was to investigate the effect A-72 cells exposure to *ST*. *ST* showed ability to penetrate A-72 (Log 10 4.14 ± 0.82) causing a significant (*P* = 0.001) reduction of cell viability (- Log 10 0.2;—12.5% Fig. 3). Moreover, *ST*’s infection (MOI 100:1 CFU/cells) caused the up-regulation of *IL6* (*P* < 0.000 1), *CXCL8* (*P* < 0.0001) and *IL15* (*P* < 0.0001) expression 1 h post-infection, while *MYD88* (*P* = 0.0242), *NFKB/p65* (*P* = 0.0414), *RAD51* (*P* = 0.0002) and *TP53* (*P *= 0.0012) were down-regulated (Fig. 4). Other genes under study were not significantly modulated.

### Sequencing of the key gene CXCR4 and TP53

The comparison of the sequences obtained by sequencing of CXCR4 and TP53 in A-72 cell line and in canine healthy and neoplastic tissue samples with sequences available in GeneBank highlighted the absence of mutations in the amplified fragments. Moreover, no differences were found in CXCR4 and TP53 sequences obatined by different passages of the cell line.

## Discussion

To date, sarcoma biology studies have been conducted in human cell lines and xenograft tumors, and there has been extremely limited progress in the non-surgical or post-surgical treatment options available to patients compared to other cancers (Dodd et al. 2010; Post 2012; Gamboa et al. 2020). In STS, however, there is a limited number of tumor cell lines available for functional testing and target validation. Data from large-scale cancer cell line studies such as the Cancer Cell Line Encyclopedia and Sanger Cancer Cell Line Project showed that < 2% of the commercially available cell lines studied are derived from STS and the majority of these belong to only one of the recognized subgroups (Salawu et al. 2016).

Therefore, animal models are indispensable tools for the study of STS because a scarcity of clinical samples makes difficult a large-scale analysis of human samples, and on the other hand, there is a growing need to establish a wider range of STS cell lines for functional testing (Dodd et al. 2010; Salawu et al. 2016).

A-72 is a continuous cell line isolated in 1980; since then, A-72 has undergone numerous passages in different laboratories in the world (Croci et al. 2011; Pratelli and Moschidou 2012). In this respect, the use of cell lines for research studies requires detailed knowledge of the purity, phenotype, species/tissues of origin, protein and gene basal expression (Geraghty et al. 2014). Therefore, the monitoring of cell lines contamination and their characterization is extremely important in order to achieve accurate conclusions. In our study, we characterized A-72 cell line focusing on the basal expression of genes involved in the innate immune response and in the cell cycle regulation; moreover, we evaluated the effect of cellular passages and monolayer aging on gene expression in order to verify the maintenance of the original phenotype characteristics. The second aim of our study was the evaluation of the A-72 ability to interact with an infectious stressor, whose the attenuated form was already explored as a novel anti-cancer therapy (Chirullo et al. 2015b; Bolhassani et al. 2017). In order to reproduce ordinary laboratory activity, cells at three different passages (104^th^, 107^th^ and 109^th^) were used.

Our results showed in A-72 the expression of all tested genes under study with the exception of *IL10* and *IL18*, in agreement with previous studies that reported no expression of those genes in this type of cells (Rutz and Ouyang 2016; Yasuda et al. 2019). However, these interleukins were expressed in the cancerous tissues included in the study; this difference could be attributed to the sample nature, being A-72 a continuous cell line while the tumor tissue is constituted by different cell types (Cancer Associated Fibroblast, T lymphocytes Macrophages e.g.).

The *CXCR4* expression tested by RT-qPCR agrees with the strong positivity at the immunocytochemical assay (Table 3 and Fig. 2). The high expression of this protein in A-72 suggests that the cell line originated from an aggressive cancer, indeed CXCR4 is known to be abundantly expressed in STS and is an independent predictor of poor prognosis and metastatic disease (Kim et al. 2011).

CD44 is a glycoprotein involved in cell–cell signalling, migration and adhesion as well as in malignant tumor initiation; moreover, it is involved in several tyrosine kinases activation such as ERBB2 and TGFβ, that have been implicated in key oncogenesis pathways, this marker is also associated with stem cancer cells in human STS (Henderson et al. 2018) and predicts worse oncologic outcomes. In our study, CD44 showed moderate level of gene expression but low protein presence in A-72 (Table 3 and Fig. 2), suggesting the stem potential maintenance of these cells. Other cell cycle regulators were investigated in terms of transcripts including TP53, ERBB2 and PTEN. TP53 plays an important role in the regulation of cell response to stress and damage (Levine 2020); ERBB2 is a member of the epidermal growth factor receptor family, acting as tyrosine kinase receptors and it is considered a potent mediator of cell growth and cancer development (Wolfson et al. 2016); while PTEN is involved in many cellular functions including cell survival, proliferation, migration and adhesion (Yan et al. 2021). Our data showed a low PTEN gene expression in A-72 while ERBB2 presented high expression level (Table 3), in agreement with a previous study conducted on human STS (Trabelsi et al. 2015). HE sample showed no expression of PTEN, this result may be explained by technical and/or biological reasons. The presence of nucleotide variants, in the PTEN sequence of the tissue, may be responsible for a mismatch between primer and template that prevents the detection of PTEN expression. Instead, taking into account a biological explanation, several sarcomas carry genetic abnormalities (deletions and mutations) in well-known tumor suppressor genes (i.e. TP53 and PTEN) which cause deregulation and loss of function of these genes. The loss of PTEN function affects important pathways implicated in cell proliferation, survival, migration, and genomic stability. A recent study on human sarcomas and STSs reported that the loss of PTEN expression is present in 38.6% of tumors and the reduction or loss of PTEN has been reported in a subset of MPNSTs, both in human and animal models (Stefano and Scambia 2019). The A-72 showed a low expression of TP53 which could be due to an altered pathway in a similar way to what has already occurred in humans (Post 2012). Concerning TGFβ and RAD51 a moderate expression was assessed, while for STAT5 a high expression was detected. This is an important finding since TGFβ is a multifunctional cell regulatory cytokine, able to modify proliferation, differentiation, tissue repair, and extracellular matrix formation. Although this protein is usually involved in cell growth suppression, it may play an important role in promoting cancer development (Massagué 2008). The expression of STAT5 could be of clinical importance considering its possible role as target of immunotherapy (Verdeil et al. 2019). RAD51 plays a pivotal regulatory role in meiotic and homologous recombination DNA repair. The over-expression of this gene has been observed in a variety of cancer and has been found associated with chemoresistance in human STS (Brown et al. 2009; Nagaraj et al. 2011; Tennstedt et al. 2013). A TP53 mutation seems to be the cause of RAD51 over-expression (Hannay et al. 2007), nevertheless in our study no TP53 mutations were detected.

Cells showed a fibroblast-like morphology confirmed by the molecular findings and the immunocytochemical results (expression of vimentin and negativity for CK), indicating the cell line mesenchymal origin supposedly referable to a perivascular wall tumor for his positivity to calponin and actin (Pérez et al. 1996; Avallone et al. 2007).

Furthermore, the involvement of cell markers essential for the interaction with bacteria was investigated. Indeed, the basal expression level of TLRs 4 and 5, belonging to the family of Pattern Recognition Receptors (PRRs), as well as LY96 and CD14, expressed by many cell types, in order to recognize bacteria and to induce the innate immune response were investigated (Velloso et al. 2015). The binding of microbial PAMPs to host cell TLRs leads to an inflammatory response, including the secretion of cytokines and chemokines. The expression in all samples of NFKB/p65, one of the major members of the NF-kB protein family, along with NOS2, MYD88, IL6 and CXCL8, implies the ability of this cell line to mount an inflammatory response. In our experiment, the treatment of A-72 with *ST* determined the up-regulation of some important proinflammatory cytokines and chemokines (IL-6 and CXCL8), and the modulation of transcription factor genes (NF-kB1, NFKB/p65, MYD88) demonstrating the activation of an inflammatory response through the NF-kB pathway (Fig. 4). The expression in A-72 of these molecules is in line with our previous results (Razzuoli et al. 2014, 2017, 2018) showing that the interaction between *ST* and A-72, as previously reported, induces CXCL8 expression, which allow *ST* to penetrate into the cells (Gewirtz et al. 2000; Stecher et al.^2007^; Chirullo et al. 2015a, b).

In this study, we also demonstrated the effect of several cell passages on A-72; this is a very important issue that must be evaluated not only during gene expression studies but also in host/pathogen interaction experiments and evaluation of anticancer therapy evaluation. Indeed, both passages and aging seem to alter the gene expression of several important genes: *NFKB/p65*, *TP53*, *TLR5* (Table 4 and Fig. 1). In particular, we observed a decrease of *CD14*, a molecule associated with LY96 and TLR4 in response to Lipopolysaccharides (LPS); suggesting a possible alteration in cell sensitivity to bacteria or LPS (Begni et al. 2005; Amadori & Razzuoli et al. 2014) after 24 h of monolayer achievement.

The variations of gene expression outlined by our results are in agreement with studies on other cell lines that demonstrated changes in basal gene expression related to cell passages (Begni et al. 2005; Amadori & Razzuoli et al. 2014). In our opinion, these variations should be considered when drawing up the experimental design.

Our results demonstrate the validity of A-72 cell line as in vitro animal model for the study of specific subtypes of human STSs (namely malignant peripheral nerve sheath tumor) and support the hypothesis of using primary STS canine spontaneous tumors, which develop in the native microenvironment of an animal with an intact immune system, for the preclinical study of new treatments.

## Conclusions

Our data highlighted the maintenance by the A-72 cell line of supposed tumor of origin (STS) phenotypical characteristics and gene expression parameters. In addition, A-72 showed the ability to respond to an infectious stressor such as *ST*. This characteristic can be exploited using the A-72 as a possible in vitro model for the preliminary assessment of novel anticancer therapeutic approaches founded on the use of the already established attenuated form of these bacteria.
